# Case Report: identification of a novel 9.159-kb deletion in a Chinese α-thalassemia family using single molecule real-time technology sequencing

**DOI:** 10.3389/fgene.2025.1669814

**Published:** 2025-11-05

**Authors:** Shulin Wu, Zonghui Feng, Fuxiang Jiang, Min Zhao, Peng Jiang, Gang Xiao, Xian Zhang

**Affiliations:** Neonatal Disease Screening Center, Huaihua City Maternal and Child Healthcare Hospital, Huaihua, Hunan, China

**Keywords:** α-thalassemia, SMRT, deletion, HBA2 gene, rare variant

## Abstract

**Background:**

Thalassemia is one of the most common monogenic disorders worldwide and classified as α-thalassemia and β-thalassemia. Conventional methods for diagnosis of thalassemia, constrained by their focus on commonly known genotypes, can easily lead to missing or misdiagnosis of rare thalassemia genotypes.

**Case report:**

We report the case of a 32-year-old pregnant woman with abnormal hematological parameters. Conventional gap polymerase chain reaction (Gap-PCR) and PCR-reverse membrane hybridization (PCR-RDB) were performed to analysis the 23 common thalassemia variants, but did not identify any pathologic variants. Next, we used PacBio third-generation sequencing platform based on single molecule real-time technology (SMRT) for this woman and her newborn and identified a novel 9.159-kb large deletion (chr16:165599-174758, GRCh38) of α-globin gene loci, which disrupted *HBA2* gene. And the breakpoints of the deletion were validated by gap-PCR and sanger sequencing.

**Conclusion:**

Our study identified a novel large deletion, which expanded the α-thalassemia gene variant spectrum and confirmed the efficiency of SMRT technology in detecting rare thalassemia variants, provided genetic counseling and prenatal intervention in clinical rare patients.

## 1 Introduction

Thalassemia represents a diverse group of inherited hematological disorders characterized by disrupting the balance of α- and β-globin chain synthesis, leading to chronic anemia and associated complications (De Simone et al., 2022; Taher et al., 2018). α-Thalassemia, primarily caused by deletions in the highly homologous α-globin gene cluster (*HBA2* and *HBA1*) on chromosome 16p13.3, presents significant challenges for molecular diagnosis. The clinical spectrum ranges from asymptomatic carriers to fatal hydrops fetalis, heavily influenced by the number and type of affected α-globin genes (Taher et al., 2018).

With the development of new technologies, an increasing numbers of thalassemia variants have been reported, with over 1000 documented to date (https://globin.bx.psu.edu/cgi-bin/hbvar). However, the traditional molecular diagnostic technologies such as Gap-PCR and PCR-RDB are mainly focused on 23 common variants (Giardine et al., 2021). Although techniques like multiplex ligation-dependent probe (MLPA) can detect structural variants, they are labor-intensive, costly, and may miss deletions whose breakpoints fall outside probe regions. Next-generation sequencing (NGS), despite its broad coverage, struggles with the highly homologous α-globin cluster and large structural variations due to short read lengths, often yielding ambiguous results (Huang et al., 2024).

The emergence of third-generation sequencing, particularly Single Molecular Real-Time (SMRT) sequencing, offers a transformative approach. SMRT, characterized with high-fidelity (HiFi) long reads enabling precise haplotype phasing and direct detection of structural variants, including large deletions and complex rearrangements, within difficult genomic regions like the α-globin cluster (Xiao and Zhou, 2020; Xu et al., 2020). To date, SMRT sequencing has proven highly effective in expanding the thalassemia variant spectrum, having identified approximately 30 novel deletion types of α-thalassemia that were previously undetectable (Chen et al., 2023; Yuan et al., 2023; Ning et al., 2024). This capability is crucial for expanding the known spectrum of thalassemia variants and improving diagnostic accuracy.

Here, we first reported a novel genotype of α-thalassemia in a Chinese family identified by SMRT technology. This case underscores the critical role of advanced technologies in elucidating rare genetic causes of thalassemia.

### 1.1 Case description

**FIGURE 1:**
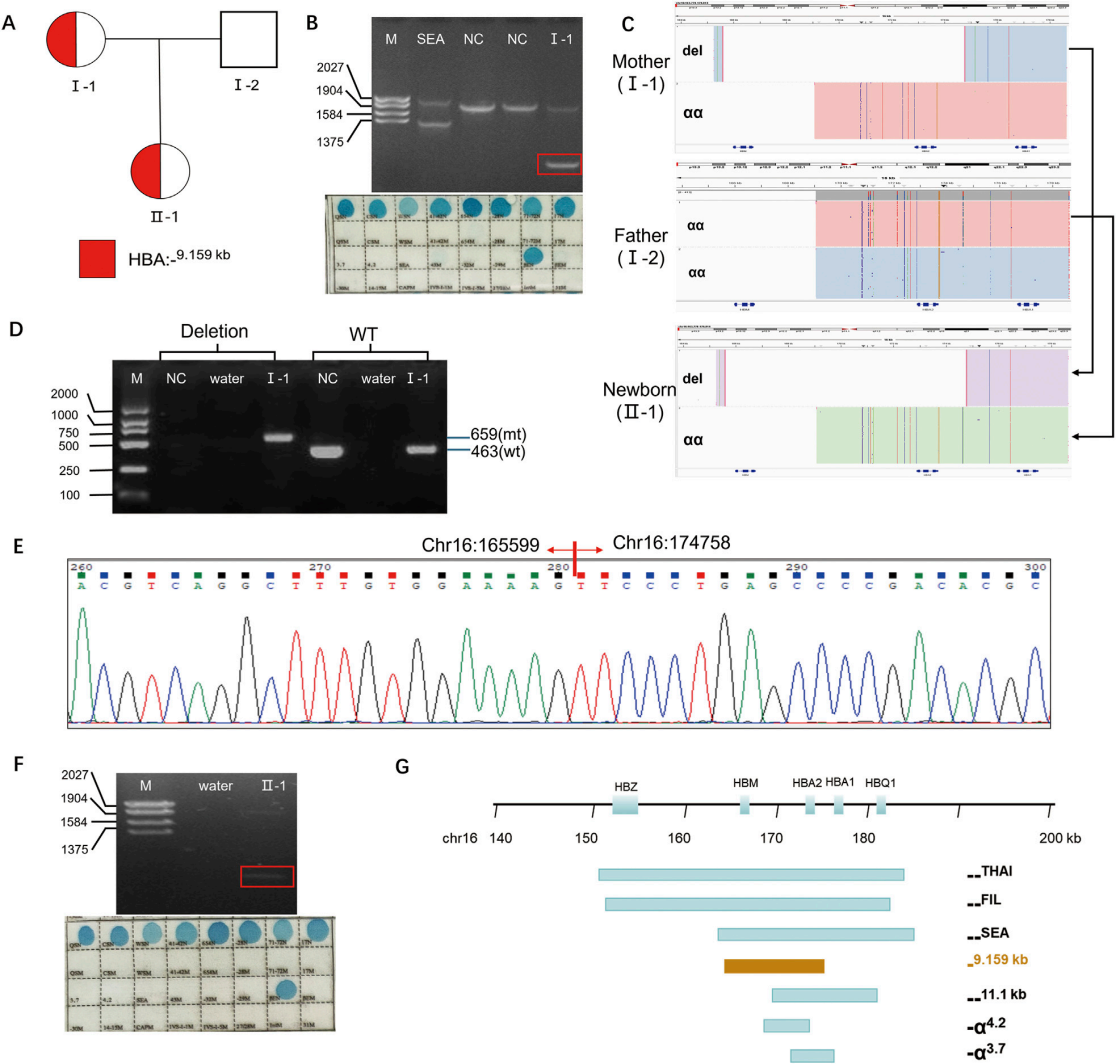
Results of the thalassemia genetic test. **(A)** Pedigree of the family. The proband (I-1) and her newborn (II-1) are indicated. **(B)** Conventional thalassemia genetic test results for the proband (I-1). *Top panel:*Gap-PCR result showing an abnormal band (rectangle). *Bottom panel:*RDB result showing no common pathogenic variants detected. **(C)** Integrative Genomics Viewer plot displayed the CCS reads of SMRT sequencing for the family. The newborn (II-1) inherited the deletion (del) allele from the mother (I-1) and the wild-type (wt) allele from the father (I-2). **(D)** Validation of the heterozygous deletion by Gap-PCR. A specific primer pair (flanking the region chr16:168803-183737, GRCh38/hg38) was used to amplify the 9.159-kb deletion. **(E)** Sanger sequencing chromatogram confirming the exact breakpoints of the deletion in the proband (I-1). **(F)** Conventional thalassemia genetic test results for the newborn (II-1). *Top panel:*Gap-PCR result showing an abnormal band (rectangle).*Bottom panel:*RDB result showing no common pathogenic variants detected. **(G)** The 9.159-kb large deletion and other common deletions were displayed in the α-globin gene cluster.Note: NC: nomal control, del: deletion, mt: mutant, wt: wild type.

A 32-year-old primigravida was referred to our hospital for genetic screening of thalassemia in May of 2024, prompted by abnormal hematological parameters at 11 weeks and 2 days of gestation. The proband showed decreased level of mean corpuscular volume (MCV) (76 fL, normal range ≥82 fL), mean corpuscular hemoglobin (MCH) (24.6 pg, normal range ≥27 pg), and iron metabolism indicators were normal (ferritin = 91.9 μg/L), indicating microcytic hypochromic anemia (Table 1). In the hemoglobin (Hb) analysis by capillary electrophoresis (CE) (Capillarys 2 Flex Piercing; Sebia, Lisses, Paris, France), Hb A2 was 2.2% (normal range 2.4%–3.5%) (Table 1), demonstrating the proband had α-thalassemia phenotype. The proband’s husband had normal hematological parameters (Table 1). Thus, the doctor recommended the proband to undergo routine genetic assay of thalassemia using Gap-PCR (Yaneng Biotech, Shenzhen) and RDB-PCR(Yaneng Biotech, Shenzhen) for 23 most common thalassemia variants, including three common deletions of α-thalassemia (--^SEA^, -α^3.7^ and -α^4.2^), three common point variant of α-thalassemia (-α^CS^, -α^QS^, -α^WS^) and 17 common variants of β-thalassemia (CD41-42 (-TCTT), IVS-II-654(C>T), −28 (A>G), CD71/72 (+A), CD17 (AAG>TAG), CD26 (GAG>AAG), CD43 (GAG>TAG), −29 (A>G), CD31 (-C), −32(C>A), IVS-I-1 (G>T), CD27/28 (+C), −30 (T>C), CD14-15 (+G), Cap+40-43 (–AAAC), int (ATG>AGG), IVS-I-5 (G>C)). Although, no common variant or deletion was identified (Figure 1B). An abnormal band in the Gap-PCR result was observed (rectangle), suggesting the presence of a rare deletional alpha-thalassemia variant. To characterize this variant, SMRT was employed. Briefly (Peng et al., 2022), the target regions were amplified by long range multiplex PCR, and the PCR products were ligated to the PacBio barcoded adapters to construct SMRT library, followed by sequencing on the PacBio Sequel II platform (PacBio, Menlo Park, CA, United States) under the circular consensus-sequencing (CCS) mode with a run time of 30 h. Raw subreads were analyzed to generate HiFi reads and then aligned to the genome build hg38. All variants including SNVs, indels, large deletions and structural variations were called and annotated according to HbVar Ithanet, and LOVD databases.

**TABLE 1:** Blood screening and thalassemia genetic analysis for the enrolled.

Case		Gender	Age	RBC (1012/L)	MCV (fL)	MCH (pg)	Hb (g/L)	HbA2 (%)	Hb Bart’s (%)	HbA (%)	HbF (%)	FER	Gap-PCR and PCR-RDB	SMRT
I-1	Mother (proband)	Female	32y	5. 12	76	24.6	126	2.2	0	97.8	0	38.9	Unidentified	HBA2 -9.159kb Het
I-2	Father	Male	39y	5.24	88.7	30.5	160	2.6	0	97.4	0	93.5	—	Normal
II-1	Newborn	Female	5d	5. 55	101.4	32.3	179	0	0.3	38.7	61	—	Unidentified	HBA2 -9.159kb Het

FER: ferritin levels, /: not detected.

Interestingly, SMRT technology identified a novel heterozygous deletion in proband, which completely disrupts *HBA2* and *HBM* genes (Figure 1C). The exact 9.159-kb deletion (chr16:165599-174758, hg38) were successfully detected by SMRT technology. And the breakpoints of the deletion were confirmed by Gap-PCR with mutant primers (forward 5′- GAA​GCT​GAG​TGA​TGG​GTC​CG-3′, reverse 5′- CCT​GTC​TGC​CAC​CCT​CTT​CTG​A-3′) designed flanking the deletion region and wild type (WT) primers (forward 5′- GAA​GCT​GAG​TGA​TGG​GTC​CG-3′, reverse 5′- CTT​TAG​GGG​CATCTGTTTACTCC-3’) (Figure 1D) and Sanger sequencing (Figure 1E). To date, more than 50 deletions involving one or both alpha globin genes have been described, the most common being the -α^3.7^, -α^4.2^, Southeast Asian (--^SEA^), Filipino (--^FIL^), and Mediterranean (--^MED^) types (Figure 1G). The breakpoints of the novel deletion are different from those deletion reported (Figure 1G).

Subsequent SMRT sequencing of the husband revealed no pathogenic variants (Figure 1C). Prenatal diagnosis was therefore deemed unnecessary. Heel prick blood collected within 5 days of birth for newborn thalassemia screening detected Hb Barts at 0.3% (Table 1), suggesting a possible silent carrier state for alpha-thalassemia. The routine method revealed an abnormal Gap-PCR band (Figure 1F). Consequently, SMRT was also performed on the newborn, which identified the same large deletion previously detected in the mother (Figures 1A,C).

## 2 Discussion

Molecular diagnosis of α-thalassemia faces significant challenges due to the genetic complexity and high sequence homology of the α-globin gene cluster (Chen et al., 2023). Traditional methods such as Gap-PCR and PCR-RDB, designed primarily to detect the 23 most common variants in the Chinese population, exhibit limited capability for identifying rare or complex variants, leading to potential missed diagnoses. Current clinical practice indicates that approximately 20%–30% of α-thalassemia cases remain genetically unresolved using conventional methods (Zhou et al., 2025). While NGS offers broader genomic coverage, its short-read nature impedes the resolution of highly homologous regions and complex structural variations (SVs), often resulting in false-positive or false-negative results (Zhou et al., 2024). In contrast, SMRT technology, characterized with long-read capability (15–25 kb), high accuracy (QV50 = 99.999%), and single-base resolution, could excel at traversing repetitive sequences and homologous regions. This enables precise identification of structural variants, point variants, and complex gene rearrangements (Chen et al., 2023; Zhou et al., 2025; Ning et al., 2023; Lou et al., 2023).

In this study, initial screening of this Chinese family using conventional Gap-PCR indicated a potential rare variant, evidenced by an abnormal band distinct from the common–SEA, –α3.7, and–α4.2 targets. The size and pattern of this band led us to speculate that it could be a primer-specific artifact, possibly an amplification product bridging the–SEA forward and–α4.2 reverse primer binding sites, hinting at an underlying large deletion. This suspicion was confirmed by SMRT technology, which precisely characterized a novel 9.159-kb deletion (chr16:165599-174758, GRCh38/hg38). Its breakpoints are located on the Alu repeat region, suggesting that the deletion may be resulted by non-allelic homologous recombination (NAHR). As a highly repetitive element in the genome, the Alu element is a hot spot for chromosomal structural variations (such as deletions and duplications) (Kim et al., 2016). Similar mechanisms are also reflected in previously reported deletions such as--^THAI^ and--^FIL^ (Eng et al., 2000). During the synthesis of alpha globin, the *HBA2* gene encodes 2-3-fold more protein than the *HBA1* gene (Liebhaber et al., 1986; Farashi and Harteveld, 2018). The deletion *HBA2* gene would have greater impact in the generation of the alpha-thalassemia phenotype, in comparison to deletion *HBA1* (Liebhaber et al., 1986). The 9.159-kb deletion only disrupts *HBA2* gene. The heterozygous carrier of this deletion was suspected to have phenotype of α-thalassemia trait similarly as -α^4.2^. Indeed, the hematological parameters of the proband and her newborn showed mild microcytic hypochromic anemia, which were well correlated with the genotype. Consequently, when the genotypes obtained from routine genetic testing are inconsistent with the hematological phenotypes, it is crucial to conduct additional genetic analysis. Hence, both hematological analysis and genetic testing need to be applied with caution to prevent false-negative results in thalassemia genetic testing. The significance of integrating various technologies to attain accurate diagnoses has been underscored by our research.

Patient perspective: The parents experienced significant anxiety after their child’s abnormal blood test, which was compounded by inconclusive results from conventional methods. The definitive diagnosis of a mild α-thalassemia variant via SMRT sequencing provided immense relief, ending a stressful diagnostic odyssey. The patient affirmed that this clarity was crucial. It explained the mild hematological anomalies in both herself and her child, alleviating concerns about other undiagnosed conditions. Furthermore, it delivered essential information for future family planning, enabling a clear understanding of the inheritance pattern and the associated reproductive risks for future generations.

In summary, this study revealed a novel 9.159-kb *HBA2* gene deletion through SMRT sequencing in a Chinese family, resulting in mild alpha + -thalassemia phenotype. This investigation demonstrates SMRT’s capability to identify novel variants that are often undetectable by conventional analytical techniques. Consequently, SMRT represents an effective and dependable method for screening thalassemia in individuals potentially harboring rare variants or complex genetic variations. Moreover, SMRT analysis should be integrated into the diagnostic pathway for ambiguous thalassemia cases to enhance diagnostic precision and the quality of genetic counseling. Carriers of thalassemia variants can utilize prenatal diagnosis and preimplantation genetic testing (PGT) services to mitigate the risk of having offspring affected by the disease.

## Data Availability

The original contributions presented in the study are included in the article/supplementary material, further inquiries can be directed to the corresponding authors.
